# A systematic review of studies on connected speech processing: Trends, key findings, and implications

**DOI:** 10.3389/fpsyg.2022.1056827

**Published:** 2022-11-29

**Authors:** Huichao Bi, Samad Zare, Ursula Kania, Rong Yan

**Affiliations:** ^1^Department of Educational Studies, Academy of Future Education, Xi’an Jiaotong-Liverpool University, Suzhou, China; ^2^Global Digital Citizenship Center, Academy of Future Education, Xi’an Jiaotong-Liverpool University, Suzhou, China; ^3^Department of English, University of Liverpool, Liverpool, United Kingdom

**Keywords:** connected speech processing, production and perception, systematic review, trends, key findings

## Abstract

Connected speech processing (CSP) is of great significance to individuals’ language and cognitive development. It is particularly crucial not only for clinical detection and treatment of developmental disorders, but also for the Foreign/second language teaching instructions. However, given the importance of this field, there is a clear lack of systematic reviews that summarize the key findings of previous studies. To this end, through searching in the scientific databases PsycInfo, Scopus, PubMed, ERIC, Taylor and Francis, and Web of Science, the present study identified 128 core CSP articles with high reference values according to PRISMA guidance and the following results were obtained through quantitative analysis and qualitative comparative synthesis: (1) The number of studies on CSP published per year showed an upward trend; however, most focused on English language, whereas the studies on other languages were comparatively rare; (2) CSP was found to be affected by multiple factors, among which speech speed, semantics, word frequency, and phonological awareness were most frequently investigated; (3) the deficit in CSP capacity was widely recognized as a significant predictor and indicator of developmental disorders; (4) more studies were carried out on connected speech production than on perception; and (5) almost no longitudinal studies have ever been conducted among either native or non-native speakers. Therefore, future research is needed to explore the developmental trajectory of CSP skills of typically developing language learners and speakers with cognitive disorders over different periods of time. It is also necessary to deepen the understanding of the processing mechanism beyond their performance and the role played by phonological awareness and lexical representations in CSP.

## Introduction

It is universally acknowledged that speech processing is the core of spoken language cognition. Only if speakers perceive phonological sounds appropriately can they establish connections between sound and meaning to achieve effective communication (Greenberg and Ainsworth, 2004). However, the speech utterances on various electronic media (e.g., film and television shows) and everyday conversations produced by native speakers are quite different from the citation form of words. The degree of these acoustic changes varies on an individual basis (Johnson, 2004). Taking English as an example, there are phonological variations as a *lingua franca* spoken around the world; for instance, the phrase “this year” /ðɪs jiə/ may be shortened as /ðɪʃiə/ (Wong et al., 2017a) and the sentence “do you have?” may be reduced to /dʒav/ (Wong et al., 2019). These phonological variations, also known as reduced forms, sandhi variation, or acoustic reductions, are generally defined as connected speech processes (CSPs), a term which refers to the changes in traditional word forms in connected speech due to articulatory and temporal constraints (Alameen and Levis, 2015). These changes occur randomly without awareness, sometimes at word boundaries, and sometimes even within words, and are difficult to predict (Ernestus, 2014). From the articulatory perspective, the function of CSPs is to promote rhyme regularity and maintain time for natural speech production (Clark and Yallop, 1995).

As one of the vital branches of speech processing research, CSP initially aroused the interest and attention of phoneticians and linguists who started to approach this phenomenon by exploring features, definitions, acoustic cues, and processing models from the articulatory and prosodic perspectives (e.g., Clark and Yallop, 1995; Shockey, 2003). One of the crucial contributions accomplished was to identify and categorize the specific types of CSPs from native speakers’ natural speech flows based on the articulatory and prosodic features such as palatalization, contraction, juncture, assimilation, flapping, vowel weakening, elision, intrusion, and glottalization (Brown and Kondo-Brown, 2006). It is apparent that the exploration of the phonetic features of CSPs in the early stages laid a solid foundation for the later interdisciplinary studies, given that the articulatory and prosodic perspectives could not generalize the CSP variants due to the use of the variety of terminologies, measurement scales, and the new research angles taken by the scholars beyond the field of linguistics. As a consequence, a more generic production and perception perspective was widely adopted for a better explanation of the entire CSPs speech processing in a broader and interdisciplinary field which may cover clinical psychology, psycho/computational linguistics, and language teaching and instruction (Ernestus, 2014; Alameen and Levis, 2015).

Production and perception, as the two important speech processing stages, are not only examined separately as independent cognitive skills, but also studied as an interrelated combination from a holistic perspective. In a broad sense, connected speech production relates to the processing of regular pronunciation features and syllable segmentation in the output process (Sardegna, 2011). Therefore, speech analysis from the production perspective provides insights into phonetic features which is more applied in the area of language instruction, screening, evaluation, and diagnosis of language/cognitive impairments and developmental disorders (Pluymaekers et al., 2005; Dennis and Hess, 2016; Ernestus et al., 2017; Wong et al., 2019; Alharbi et al., 2021). By contrast, connected speech perception is closely associated with listening comprehension emphasizing top-down processes more than bottom-up ones (Field, 2003). Therefore, CSP studies from perceptual perspectives were more focused on perceptual error analysis (Wong et al., 2017a,b, 2021b; Bhatt et al., 2021), ESL/EFL instructions (Chen et al., 2021), and early detection of cognitive decline in thought and mental disorders, such as Alzheimer’s disease (e.g., Voleti et al., 2019).

Although native speakers can efficiently process connected speech, the randomness and complexity may primarily cause perceptual and comprehensive difficulties for many FL/SL learners as well as those with cognitive impairments and deficits (Ernestus et al., 2017; Behroozmand et al., 2018; Wong et al., 2019). Given the importance mentioned above, scholars conducted a large number of empirical studies and experimental reports. However, a few review articles have only focused on the groups with specific disorders (Boschi et al., 2017; Kave and Goral, 2018; Voleti et al., 2019; de la Fuente Garcia et al., 2020), or a particular connected speech subtype (Veselovska, 2016) and specific category (Kave and Goral, 2017; Mason and Nickels, 2022), and they are thus unable to reveal the whole spectrum of the current literature. The only two pieces of research that provide a more comprehensive overview of connected speech studies were restricted to the typically developing group from the linguistic perspective (Ernestus, 2014; Alameen and Levis, 2015). They neither cover the CSP studies on speakers with developmental disorders nor do they include empirical findings from the last 8 or 9 years. Many empirical results highlight that recent findings have not been sufficiently applied to practice. For example, the detection and treatment of cognitive decline in production has not been effectively applied to clinical practice (de la Fuente Garcia et al., 2020), and the teaching instructions on connected speech in EF/FL classrooms lack effective theoretical support and practical guidance (Wong et al., 2019). Obviously, there is a lack of complete, holistic, and systematic reviews to sum up what has been accomplished over the past decades and what needs to be further explored in the future. It is unclear what distribution rules and differences exist in the perception and production perspectives of connected speech among different groups. Whether the current research results can well reveal the processing mechanism and learning models behind the CSP ability needs verification.

Therefore, the systematic sorting of existing research findings is of great significance for researchers to better understand the defects and deficiencies of existing research and to carry out practical intervention and practice. Specifically, this may provide unique insights into enriching psycholinguistic theories and speech processing models for research, detecting cognitive functioning decline and treatment of developmental disorders for clinical practice (Behroozmand et al., 2018), and developing listening comprehension and cognitive decoding skills of FL/SL learners for education purposes. Moreover, it is also claimed to contribute to automatic speech recognition and digital speech processing through the analysis of common articulatory features and voice normalization of different speakers (Furui, 2001; Rabiner and Schafer, 2007).

### Present study

This study adopts a systematic review method to summarize the general trends and key findings of CSP studies among typically developing speakers and those with developmental disorders and, more importantly, it reflects on the contributions and implications of previous studies from a heterogeneous, multilingual, and interdisciplinary perspective. The present study intends to address the following three questions:

(1) What are the general characteristics and longitudinal trends of studies on CSP? (2) What are the key findings of the studies on CSP? (3) Based on the results for RQs 1 and 2, and considering the limitations discussed in the studies under analysis, what aspects of CSP should be further explored in the future?

## Materials and methods

### Database and search strategy

Given the interdisciplinary nature of studies on CSP, the target databases were chosen to cover the fields of psychology, cognitive behavior, language education, applied linguistics, psycholinguistics, and computational linguistics. The domain terms searched for in the relevant title, abstract, or topic in these databases were “connected speech processing,” “connected speech perception,” and “connected speech production; some alternative terms were also adopted as the search terms. To be specific, synonyms of the term “connected speech” such as reduced forms, casual/natural/everyday speech, daily conversations, sandhi variation, acoustic reduction, phonological variants in spontaneous speech, as well as any identified types of connected speech processes (e.g., linking, elision, assimilation, juncture, flapping, and liaison) were also searched. In addition to the term “processing,” the search terms perceptual errors, productive skills, acquisition, processing skills, and listening performance/comprehension were added to include as much literature as possible. All search terms based on relevant literature on connected speech processing were included in the six electronic databases (PsycInfo, Scopus, PubMed, ERIC, Taylor and Francis, Web of Science), in January 2022 and again in August 2022. The search period was not limited and aimed to include as much available literature with abstracts in English as possible in several fields.

### Data collection

**Figure 1:**
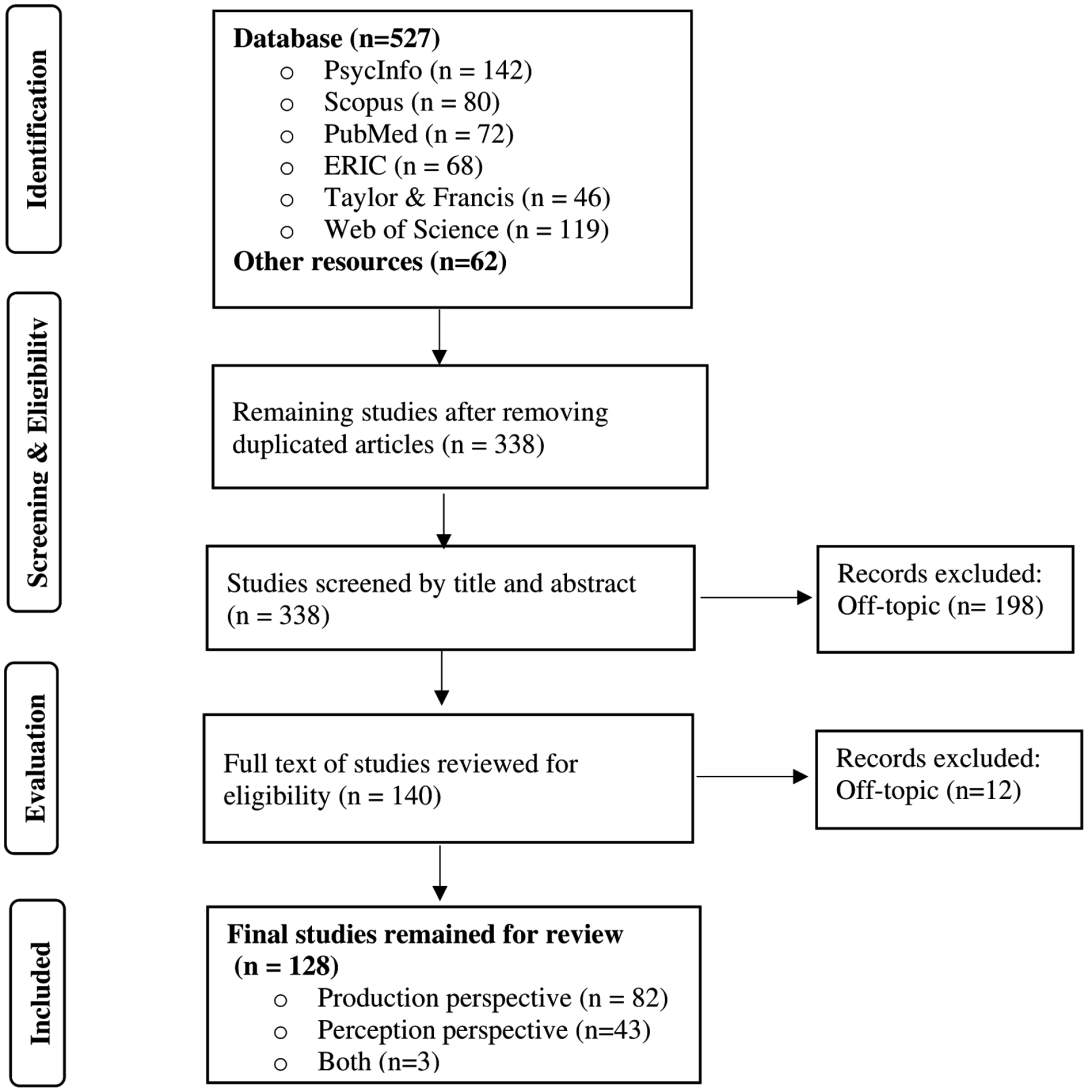
PRISMA flowchart of review process.

As shown in Figure 1, a total number of 589 peer-reviewed publications were primarily retrieved from six databases. After removing 251 duplicates, there were 338 publications to be further reviewed. After an examination of the titles and abstracts for eligibility, 198 off-topic articles were excluded since they were not focused on connected speech, and then the full texts of 140 articles were screened again for the second round of evaluation, which, furthermore, excluded 12 off-topic pieces of literature. Ultimately, a total number of 128 articles were subjected to the final analysis.

### Data analysis

The following information from each screened publication was summarized in Microsoft Excel for quantitative analysis and qualitative comparative synthesis (Table 1).

**Table 1:** Subcategories of research questions.

Research questions	Subcategories
RQ1	Reference: year of publication (in text)
	Types of languages: English connected speech = 1; other languages = 2 (also in text)
	Perspectives of connected speech processing: perception = 1, production = 2, both = 3
	Methodology: qualitative = 1, quantitative = 2, mix = 3
	Measurement (in text)
	Subject age by years: toddlers (birth-to-3-year) = 1; early Childhood (3–6-year) = 2, childhood (6–12-year) = 3, adolescent (12–18-year) = 4, young adulthood (18–35-year) = 5; middle adulthood (36–55-year) = 6, older adulthood (56-year and older) = 7
	First language: English as L1 = 1, other languages as L1 = 2 (also in text), mix group = 3 (also in text)
	Subject: typically developing = 1, developmental disorders = 2, mix group = 3
RQ 2	Open coded strategy and content analysis for the main findings, limitations, and implications of studies of connected speech processing

In order to ensure inter-rater reliability, two established scholars in the field of psycholinguistics and educational psychology were invited to code the literature separately. The Cohen’s Kappa coefficient value was found to be higher than 0.80, presenting an almost perfect agreement between the two coders.

## Results

### Research trends on CSP

#### Overview: Types of languages, distribution of studies by years, and research methods

**Figure 2:**
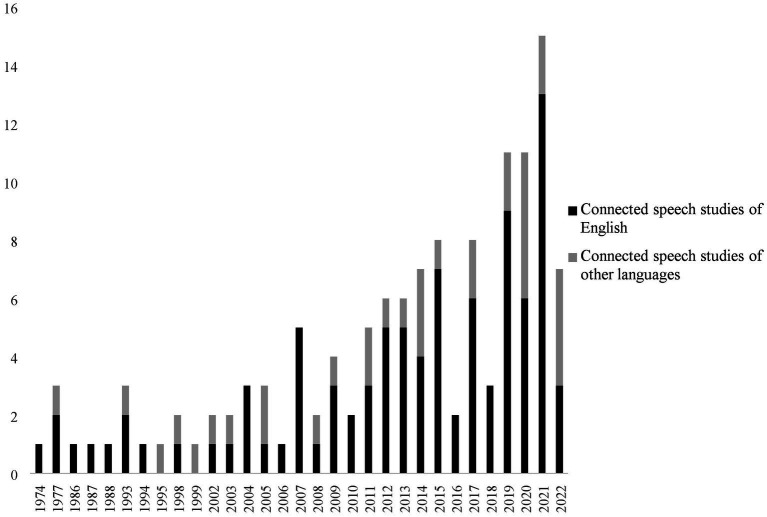
The number of reviewed articles of connected speech processing of years.

Overall, 128 peer-reviewed articles on CSP published between 1974 and 2022 were analyzed. As shown in Figure 2, the number of studies followed an overall ascending trend, starting to increase significantly in 2011, and reaching the peak with 15 publications in 2021. In addition to this, these studies were primarily concentrated on English speakers (72.7%), while only 27.3% of studies involved other languages. A total number of 15 languages were explored, namely, French (Hesling et al., 2005; Girard et al., 2008; Burki et al., 2011; Kennedy and Blanchet, 2014), Korean (Mitterer et al., 2013; Kim et al., 2022), Greek (Kambanaros, 2014), Mitterer and McQueen, 2009), Dutch (Ernestus et al., 2017), Norwegian (Kirmess and Lind, 2011), Telugu (Hivaprasad and Sadanandam, 2020), Cantonese (Yiu et al., 2002), Persian (Daneshi et al., 2020), Finnish (Alexandrou et al., 2017), Bengali (Bose et al., 2022), Spanish (Guzman et al., 2021; Gonzalez-Alvarez and Sos-Pena, 2022; Lofgren and Hinzen, 2022), Portuguese (Brinca et al., 2014; Sampaio et al., 2019), Swedish (Alves et al., 2020; Strombergsson et al., 2020), Mandarin (Tsai et al., 2012), and Italian (e.g., Cerrato et al., 1998; Leoni and Cutugno, 1999). In addition to English, studies on Italian connected speech were more abundant than that of other languages. Specifically, scholars explored the unique features of Italian connected speech such as sound patterns of various local accents (Bertinetto and Loporcaro, 2005), typical phonological variation (Vietti, 2019), strength-based faithfulness and the sibilant /s/ (Baroni, 2015), vowel system and reduction phenomenon (Leoni et al., 1995; Romano, 2020); influential factors such as the visual and prosodic information to processing Italian connected speech (Cerrato et al., 1997); and the wavelet-transform systems of Italian connected speech (Cutugno and Maturi, 1993). There were also comparative studies between Italian and English regarding automatic natural speech syllabification (Petrillo and Cutugno, 2003) and speech production differences (Canu et al., 2020).

Among the 128 articles, there were seven review articles, and the remaining were reports based on empirical studies. Consistent with our assumption, quantitative methods were predominantly adopted in these studies, while only few employed qualitative or mixed approaches, such as error rate analysis, or presented case and exemplar studies. The common connected speech production measures used for speakers with developmental disorders included behavioral tasks (e.g., story retelling, picture description, word imitation, concurrent commenting, and free conversation), psychiatric rating scales (De Prete et al., 2021), standardized tests (Kirmess and Lind, 2011), corpus analysis, Voxelwise Lesion-Symptom Mapping (VLSM; Stark et al., 2019), and functional Magnetic Resonance Imaging (fMRI; Narayana et al., 2020). The data drawn from these instruments were processed by various statistical techniques ranging from the K-means algorithm, SPSS, and PRAAT speech software to spectral/cepstral analyses (Bose et al., 2022) for a more accurate and comprehensive evaluation of speech rate, dysfluencies, syntactic, lexical, morphological, and semantic malfunctions.

In contrast with the studies on speakers with developmental disorders, perception measures were more employed in the studies of typically developing groups to explore the underlying phonological representations of connected speech perceived during daily conversations. These measures included connected speech perception tasks such as auditory lexical decision task, stimuli decision task, picture pointing task, phonetic inventory and word shape analytical task (Casilio et al., 2019), corpus analysis (e.g., French corpus of radio-broadcast speech; Burki et al., 2011), repetitive priming task (Lo Casto and Connine, 2011), eye-tracking (Poellmann et al., 2014), and magnetoencephalography (MEG; Alexandrou et al., 2017). In addition to the perception measures mentioned above, a small number of studies used connected speech output tasks (e.g., reading task, dialog audio collection) and corpora (e.g., Buckeye Cos of conversational speech; Gahl et al., 2012) to analyze different output characteristics and influencing factors among normal speakers.

#### Characteristics of sampling: Age, first language, and developmental disorders

As shown in Table 2, the subjects selected in the existing CSP studies were mostly adults (88.1%; Dennis and Hess, 2016; Wong et al., 2019; Chen et al., 2021); only few focused on children, among which four studies were on toddlers (Thompson and Howard, 2007; DeVeney and Scheffel, 2019; Daneshi et al., 2020), five on pre-schoolers (Camarata, 1993; Iacono, 1998; Girard et al., 2008; Kambanaros, 2014; Tang et al., 2019), one on primary school children (Howard, 2013), and two on adolescents (Musfirah et al., 2019; Wong et al., 2020). The rest were carried out with a wide age range, mainly with groups with developmental disorders; for instance, 20–85-year-old sample with neurogenic communication disorders (Fromm et al., 2021), 9–16-year-old children with speech impairment (Howard, 2004), 21–69-year-old adults with Parkinson’s disease (Lee et al., 2019), 2–10-year-old children with Fragile X Syndrome or Down Syndrome (Barnes et al., 2009), 19–74-year-old patients undergoing left hemisphere resective surgery (McCarron et al., 2017), and 4–8-year-old siblings with hearing loss (Skoruppa and Rosen, 2014).

**Table 2:** Summary of the demographic information of participants in reviewed articles.

	Number (%)
**Age range**	
Toddlers (birth-3 year)	4 (3.6)
Early childhood (3–6-year)	5 (4.5)
Childhood (6–12 years)	1 (0.9)
Adolescent (12-18-year)	2 (1.8)
Young adulthood (18–35)	39 (35.5)
Middle adulthood (36-to-55-year)	22 (20.1)
Older adulthood (56-year and older)	8 (7.2)
Mixed/across age group	29 (26.4)
**Languages**	
Native group	102 (79.7)
Non-native group	18 (14.1)
Mixed group	8 (6.2)
**Developmental disorders**	
Typical developing	79 (61.7)
Disordered	39 (30.5)
Mixed group	10 (7.8)

The results also indicated that the majority of subjects were native speakers (79.7%), whereas the studies on non-native speakers began to appear in 2011, and comparative studies of native and non-native speakers only emerged more recently in 2016. As presented in Table 3, a total of 23 papers were empirical studies focusing on non-native speakers; only one involved speakers with developmental disorders (Kambanaros, 2010); five papers tested both native and non-native speakers, and four with mixed native language backgrounds (Euler, 2014; Shi, 2014; Ernestus et al., 2017; Nijveld et al., 2022). Similar to the overall characteristics of the subjects, except for a small number of elderly (Kambanaros, 2010) and adolescent subjects (Musfirah et al., 2019; Wong et al., 2020), most of the subjects of non-native studies were between 18 and 25 years of age, which suggests that these subjects were young adults who may have had many years of FL/SL learning experience. It is apparent that the CSP studies on early childhood and adolescence, also known as the sensitive or critical period for language development (Singleton, 2005), were relatively rare except for the study by Tang et al. (2019) which only included preschool children as the control group to compare with adult speakers.

**Table 3:** Studies of non-native sample’s connected speech processing.

	***n***	Age range	References
**English as L2 (*n* = 22)**			
Greek-English	1	60–84	Kambanaros (2010)
Mandarin-English	5	19–20	Liang (2015)
		20.8–21.2	Wong et al. (2017a)
		18.2–21.2	Li and Gollan (2018)
		3–5 and 19–25	Tang et al. (2019)
		18–20	Chen et al. (2021)
Cantonese-English	4	18.5-20.9	Wong et al. (2017b)
		15–16	Wong et al. (2020)
		17.47 (Mean)	Wong et al. (2021)
		19.9–20.4	Wong et al. (2019)*
Persian-English	2	18-30	Ahmadian and Matour (2014)
		18–25	Ashtiani and Zafarghandi (2015)
Dutch-English	3	19.9–24.4	Mulder et al. (2015)*
		18.9–24.5	Felker et al. (2019)*
		18–28	Mulder et al. (2022)
Finish-English	1	20–61	Kakouros and Rasanen (2016)*
Bahasa Indonesia-English	1	17–18	Musfirah et al. (2019)
Turkish-English	1	18–20+	Demirezen (2016)
Mixed languages	4	18–21.6	Ernestus et al. (2017)
		21–22	Nijveld et al. (2022)
		23–54	Shi (2014)
		20–40+	Euler (2014)
**Dutch as L2 (*n* = 1)**			
Mixed languages	1	19–53	Ernestus et al. (2017)*

*Comparative studies between native group and non-native group.

#### Research perspectives: Connected speech production and perception

As an interdisciplinary topic, the focused research perspectives vary in different periods. In the last century, the phenomenon of CSPs in speakers’ everyday speech initially caught the attention of phoneticians and linguists who started with the investigation of the acoustic characteristics (Lass, 1984), phonetic features (Cohn, 1993), functions (Clark and Yallop, 1995), syllable segmentation cues (Nakatani and Dukes, 1977), and pronunciation paradigms (Levis, 2005) of connected speech from the articulatory and prosodic perspectives. Besides, CSP studies were expanded to a broader linguistic field exploring the processing models from perception to production (e.g., TRACE Model, connectionist model of speech perception; McClelland and Elman, 1986; Norris, 1994). On top of these findings on features and speech segmentation rules, linguists named typical processes and classified specific categories of CSPs such as elision and flapping (Alameen and Levis, 2015).

Subsequently, based on a more comprehensive understanding of the common phonetic features and regulations in typically developing native speakers’ connected speech, studies on CSP tend to be more interdisciplinary. It is worth noting that the articulatory, prosodic, and perception perspectives of CSPs are not able to cover the entire speech processes and the interdisciplinary studies on CSP; therefore, linguistics mainly categorized CSPs studies from the perception and production perspectives in the reviews (e.g., Ernestus, 2014; Alameen and Levis, 2015). Firstly, clinical psychologists recognized that different disorders might exhibit specific patterns of linguistic deficits from the production perspectives (Drummond et al., 2015). Thus, they extended the target participants from the typically developing population to the early identification and characterization of disorders, especially neurodegenerative diseases and cognitive decline (Boschi et al., 2017). Secondly, CSP has gradually attracted the attention of psychologists, educators, and cross-language researchers since it may cause difficulties for second language learners’ listening in the perception process of connected speech. For example, there are studies on the production and perceptual difficulties, error analysis of FL/SL learners (e.g., Wong et al., 2021), and influential factors (e.g., Wong et al., 2017b). Thirdly, recent studies on linguistics also expand from the first language to the second language including contrasts, similarities, and the transfer of phonological features between two languages (Wong et al., 2019), comparing the production differences of phonetic features between native and non-native speakers (e.g., Canu et al., 2020), and analyzing the first-language phonotactic constraints impact on the second language connected speech perception and listening performance (e.g., Erestus et al., 2017). Recent CSPs studies aim to develop effective SL/FL CSPs teaching instructions and treatment for cognitive decline of developmental disorders.

This study systematically analyzed literature from the perception and production perspectives, consistent with the well-recognized categorization of essential perspectives in other reviews. The analysis result shows that the connected speech production studies (*n* = 82) greatly outnumbered those on perceptions (*n* = 43). Only three studies investigated both production and perception (Ernestus, 2014; Liang, 2015; Alexandrou et al., 2017). However, the sampling across these two domains demonstrates an uneven distribution. Specifically, early research on phonetics focused on normally developing native speakers from the articulatory perspective with little reference to FL/SL learners and those with specific disorders. Later, in the more interdisciplinary studies that followed, the subjects of connected speech production studies were dominated by native speakers and speakers with developmental disorders whereas most perception studies selected typically developing groups and non-native speakers as the subjects. In addition, the most frequently examined developmental disorder relating to CSP was aphasia (Conroy et al., 2009; Wilson et al., 2010; Herbert et al., 2012; Croot et al., 2014; Casilio et al., 2019). The other types of disorders were speech impairment (Camarata, 1993; Howard, 2004, 2013; Alves et al., 2020), cognitive impairment (Kim et al., 2022), vocal dysfunction (Brinca et al., 2014), Parkinson’s disease (Lee et al., 2019; Alharbi et al., 2021), Down Syndrome (Iacono, 1998), adductor spasmodic dysphonia (Kave and Goral, 2018), Alzheimer’s disease (Evans et al., 2021; Bose et al., 2022; Lofgren and Hinzen, 2022), voice disorders (Sampaio et al., 2019); hearing loss (Daneshi et al., 2020), and behavioral dysphonia (Guzman et al., 2021).

Unlike production studies, the subjects of perception research were mainly typically developing individuals, with only five articles focusing on speakers with developmental disorders including hearing impairment (Cox et al., 1988), developmental speech impairment (Howard, 2004), Fragile X Syndrome or Down Syndrome (Barnes et al., 2009), aphasia (Casilio et al., 2019), and Cerebral Palsy (Mahr et al., 2020). Another noteworthy trend is that since 2012, there has been a growing body of comparative studies on connected speech production among speakers with different developmental disorders, e.g., comparative studies of semantic dementia vs. Alzheimer’s disease (AD; Sajjadi et al., 2012), primary progressive aphasia vs. AD, and progressive supranuclear palsy vs. Parkinson’s disease (Beales et al., 2018; De Prete et al., 2021). Several studies compared connected speech production of normal groups with that of speakers having a specific impairment, e.g., AD vs. normal elderly (Ahmed et al., 2013), children with specific language impairment vs. normal groups (Kambanaros, 2014). Only one study compared the perceptual skills of children with hearing impairment and children with normal hearing focusing on the assimilation of the coda /t/ and /n/ in English (Skoruppa and Rosen, 2014).

### Key findings of the studies on CSP

#### CSP of typically developing speakers

A large number of studies on typically developing speakers investigated the influential factors affecting connected speech perception. These factors include speech rate (Dilley and Pitt, 2010), semantics (Alexandrou et al., 2017), phonological skills (Wong et al., 2017a), speaker differences, degree of prosodic information (Hesling et al., 2005), probabilistic speech events (Lo Casto and Connine, 2011), word predictability, position in the utterance (Burki et al., 2011), word frequency (Ranbom and Connine, 2007), and accents (Bhatt et al., 2021). Native language ability, exposure time, and meta-phonological awareness were also found to have explicit and implicit impacts on connected speech perception in early childhood (Girard et al., 2008). Moreover, a significant two-way interaction was identified between connected speech perception and production (Mitterer and McQueen, 2009).

With regard to connected speech production, typically developing speakers demonstrated steady progress in their processing capability. Unlike 90% of children who could master 90% of single words by the age of six, 3–10-year-old native speakers presented a wider range of progression at mastery levels of 50, 75, and 90% (Glaspey et al., 2021). It was also revealed that connected speech production was affected by various factors including speech rate (Ernestus, 2014), utterance length, noise condition (Huber, 2007), word frequency (Pluymaekers et al., 2005), contextual predictability, and phonological neighborhood density (Gahl et al. al., 2012). Besides, significant individual differences in connected speech production were evidenced between the elderly and younger groups. Specifically, the elderly native speakers used more irregular and atypical connected speech variants (Dennis and Hess, 2016), while the younger ones could not spontaneously produce the close juncture as the elderly did (Thompson and Howard, 2007). The context was argued to be the main cause for this difference (Kave and Goral, 2017). Some studies using fMRI and MEG technology intended to explore the processing mechanisms of connected speech production from a neuro-linguistic perspective. The results indicate that the right hemisphere of the brain played a vital role in continuous speech production (Alexandrou et al., 2017). In parallel with neuro-linguistic evidence, empirical findings from the studies of computational linguistics and artificial intelligence revealed the restricted functions of current automatic speech recognition systems. It was suggested that the most effective solution to cope with the deficits was to develop a more comprehensive speech database (Hivaprasad and Sadanandam, 2020) and optimize computer speech recognition models (Bhatt et al., 2021) in order to identify speech variations in a more intelligent, accurate, and exhaustive manner.

#### CSP of speakers with developmental disorders

The CSP research on non-typically developing groups concentrated on the role of CSP in the classification, identification, and diagnosis of various developmental disorders. Existing studies on cognitive disorders found that information units (Kim et al., 2022), pause rate and pausing to the syntactic positions (Lofgren and Hinzen, 2022), low tone to high tone ratio (Tsai et al., 2012), and deficit of CSPs (Evans et al., 2021) were effective indicators to judge the degree of cognitive decline in Alzheimer’s disease. In terms of voice disorders, connected speech data was confirmed to be one of the criteria for clinical aphasia grading (Fromm et al., 2021). Moreover, concurrent commenting was proved to be effective in promoting connected speech production in patients with dysphonia (Alves et al., 2020), while phonological skills were recognized as a significant factor affecting the connected speech production in children with Down syndrome (Iacono, 1998). Even though connected speech production was manifested in different types of deformities for people with cochlear implantation disorder, there were no significant differences among the patients with different types of malformation (Daneshi et al., 2020). Similarly, there were no significant differences in the total number of verb tokens and verb types produced in connected speech between typically developing children and children with specific language impairment; therefore, verb deficits were not recognized as discriminant indicators (Kambanaros, 2014).

Few studies examined connected speech perception among speakers with developmental disorders. For instance, Barnes et al. (2009) found that intelligibility in connected speech can discriminate different types of fragment X syndrome. In addition, Cepstral Peak Prominence was a practical approach to measure the levels of hoarseness in the connected speech of speakers with voice disorders (Halberstam, 2004). More recently, the auditory-perceptual rating was reported to be a reliable method to analyze the perception skills of connected speech in patients with aphasia (Casilio et al., 2019).

#### CSP of FL/SL speakers

Compared with native speakers, FL/SL learners exhibited a certain degree of processing difficulty in connected speech, both at perception and production levels (Liang, 2015; Wong et al., 2021). Unexpectedly, this was also found to apply to advanced second language learners (Ernestus et al., 2017). Several factors were identified to exert a direct or indirect impact on FL/SL speakers’ CSP. At the perception level, these factors include subtitles (Wong et al., 2020), phonological ability (Wong et al., 2017a), native language pronunciation rules (Ernestus et al., 2017), semantics (Shi, 2014), the familiarity of the CSPs (Kennedy and Blanchet, 2014), and different sound environments (Wong et al., 2017b); at the production level, exposure time (Ashtiani and Zafarghandi, 2015), the phonological overlap of cognates (Li and Gollan, 2018) as well as the differences between the first and second language (Wong et al., 2019) were reported to be significant factors. Furthermore, intervention studies showed that targeted phonological training (Ahmadian and Matour, 2014; Euler, 2014) and listening practice (Musfirah et al., 2019) were conducive to improving L2 learners’ connected speech perception and production.

One study, using a perceptual judgment task, investigated children’s adaptability to differentiate phonological variants of their native language, thereby revealing the existence of abstract phonological representations in native language speech perception (Tang et al., 2019). A few empirical studies with priming and brain response (EEG) experimental design also confirmed the importance of mental lexical representations in CSP among non-native speakers. The results obtained from auditory identity priming experiments suggest that the exemplars might differ between native and non-native speakers’ speech comprehension processes (Nijveld et al., 2022). However, it remains to be investigated whether there would be similar or different types of representation for phonological variants among FL/SL learners. Besides, most of the aforementioned studies investigated the CSP factors through behavioral tests, which, to a large extent, restricts a meticulous probe into the underlying mechanism of connected speech, thus limiting the effectiveness of the CSP intervention and instruction model (Mulder et al., 2022; Nijveld et al., 2022).

## Discussion and implications

Through a systematic review of 102 peer-reviewed publications from PsycInfo, Scopus, PubMed, ERIC, Taylor and Francis, and Web of Science, this study summarized the research trends and key findings of CSP studies from a heterogeneous, multilingual, and interdisciplinary perspective. Key findings are summarized and discussed below with particular regard to limitations of existing research and the aspects of CSP that should be further explored in the future.

First of all, in spite of an overall increasing trend in the number of publications over the past decades, existing studies primarily focused on native English speakers as opposed to the speakers of other languages. In particular, there is a lack of studies on native Chinese and Indian speakers, who account for more than one-third of the world’s population (Coole, 2018). Although English is spoken as the world’s *lingua franca*, inadequate research on other languages is definitely disadvantageous for a comprehensive summary of universal laws and characteristics of CSPs. Therefore, future studies should target the speakers of other languages, especially logographic languages like Chinese to enlarge the scope of the research samples so as to enhance the understanding of the CSP mechanisms in a much wider range. In addition, the majority of the subjects of existing studies are adults, with very few focused on younger speakers and SL/FL learners in early childhood. Although empirical evidence has shown that CSP was influenced by multiple factors such as semantic, subtitling, and environmental and phonological abilities (Ernestus et al., 2017; Wong et al., 2021), very little is known about the relationship between CSP of first/mother language and that of foreign or second language. Whether there would be any cross-linguistic transfer among bilinguals and FL/SL learners requires further investigation as well (Nijveld et al., 2022).

Another interesting finding is related to the research perspective. As mentioned earlier, with regard to the different CSP stages, the number of production studies exceeded that of the perception ones. There was also an uneven distribution of research subjects at different stages, generally with the former mostly carried out among the group of native speakers and developmental disorders while the latter primarily involved typically developing FL/SL learners. An even more intriguing discovery is that production studies were more likely to compare non-typically developing speakers with normal groups, while the perception studies were inclined to contrast native and non-native speakers. The possible reasons might lie in the fact that the focus of the CSP studies transferred from the phonetic features of native speakers’ speech to the role of CSP in the diagnostic criterion and evaluation of treatment effects on developmental disorders such as Alzheimer’s disease, Down syndrome, and Aphasia. Therefore, the outward behaviors of speech output became exceptionally crucial as acoustic features and clinical clues to be identified and examined through connected speech production. More recently, due to the acceleration of globalization and internationalization as well as the increasing demands on cross-cultural communication (Sanchez-Hernandez and Baron, 2022), the impact of CSP on FL/SL speaking and listening comprehension began to receive much more attention, thus leading to a shift of research focus from production to perception. Accompanied by this shift was the change of research subject from native speakers with developmental disorders to normal FL/SL speakers. Apparently, the research perspective and objective on connected speech have been regulated by the demand for social and economic development.

Thirdly, from the research method point of view, the CSP measures varied with different research subjects. For speakers with developmental disorders, the most commonly adopted instruments include phonological output tasks, standardized tests, corpus analysis, VLSM (Stark et al., 2019), and EEG to help identify, classify, and diagnose developmental disorders from a neuroscientific and clinical perspective. In contrast, the measures for typically developing speakers were primarily behavioral tests such as phonological perception tests, reading tasks, dictation tasks, or based on corpus analysis. Only a few studies employed priming and magnetoencephalography in an attempt to probe into the function of the brain (Alexandrou et al., 2017) or the effect of word frequency and the phonological context in connected speech perception or production (Lo Casto and Connine, 2011). In other words, the conclusions of most existing studies on normal speakers were mainly drawn from the behavioral analysis with a lack of data related to the mental lexicon and phonological representations measured and presented by reaction time, eye movement, or electroencephalogram. As a consequence, mixed methods which can integrate quantitative and qualitative research paradigms as well as behavioral, cognitive/neuroscientific, and artificial intelligence techniques (Bhatt et al., 2021) are strongly recommended for future research in order to acquire more converging evidence from both typically and non-typically developing groups, thus leading to further exploration of the inner processing mechanisms behind various types of phonological processes. At the same time, constructing more connected speech corpora, especially the bilingual, multilingual, and parallel corpora involving children and adults with languages other than English is exceptionally crucial and pivotal. Only by doing so can we triangulate or verify what has been found in a more enriched and diversified language and cultural contexts for the sake of optimizing the existing theoretical speech processing models through the increase of validity and reliability of the current research findings.

The most noteworthy finding that needs to be pointed out is the scarcity of longitudinal and even cross-sectional studies which can follow the developmental trajectories of CSP skills. Moreover, the studies targeting preschool and elementary school children during critical and sensitive periods of language learning are extremely rare. As a result, there is hardly any way to know how CSP skills progress across different developmental stages, what characteristics manifest in each stage, and whether there would be any gender and cultural differences or interactions. Besides, previous studies have specified that the mental representation of phonological variants in connected speech directly affects listeners’ speech perception (Mulder et al., 2022). However, how these phonological variants are perceived, activated, stored, and retrieved by different age groups, whether the representations vary between different mother tongues or FL/SL proficiency levels, and how CSP skills are associated with language experience and cognitive maturity remain unclear. There is some evidence that suggests native and non-native speakers present different exemplars in connected speech perception (Nijveld et al., 2022), but whether abstract representations (Tang et al., 2019) or hybrid models may also exist among speakers with different language learning backgrounds is still a controversial topic (Ernestus, 2014; Bhatt et al., 2021). To clarify this controversy, more longitudinal and cross-sectional studies need to be performed to scrutinize the growth rate of CSP skills over different periods for a complete and in-depth understanding of the dynamics between the CSP and learning environment.

## Conclusion

This systematic review presents a detailed analysis of the general trends, key findings, and future research implications based on CSP studies. It primarily yields the following findings: (1) In spite of an overall increase in studies on CSP over the past decades, the majority of them focused on the English language, with a clear lack of studies on other languages; (2) for typically developing speakers, CSP skills were affected by multiple factors, most frequently investigation of which include speech speed, semantics, word frequency, phonological skills, and speaker differences; (3) CSP processing deficits and difficulties were recognized as significant predictors and indicators of various developmental disorders; (4) the studies on connected speech production greatly outnumbered those on perception. Most of the research was carried out on native speakers than on non-native speakers, and the latter were largely limited to college students or adult learners; (5) almost no longitudinal studies were conducted to explore the developmental trajectory of CSP skills of both native and non-native speakers. Moreover, the research on the phonological representations and processing mechanisms of connected speech needs to be strengthened due to the existing controversy of CSP representation models.

## Data availability statement

The raw data supporting the conclusions of this article will be made available by the authors, without undue reservation.

## Author contributions

HB and RY conceptualized and planned the paper and analyzed the results. HB conducted the search. SZ and UK provided critical feedback on the content of the manuscript. The preparation of the manuscript was supported by HB, SZ, UK, and RY. All authors contributed to the article and approved the submitted version.

## Conflict of interest

The authors declare that the research was conducted in the absence of any commercial or financial relationships that could be construed as a potential conflict of interest.

## Publisher’s note

All claims expressed in this article are solely those of the authors and do not necessarily represent those of their affiliated organizations, or those of the publisher, the editors and the reviewers. Any product that may be evaluated in this article, or claim that may be made by its manufacturer, is not guaranteed or endorsed by the publisher.
